# A case of portal venous gas after obstructive transverse colon cancer surgery

**DOI:** 10.1186/s40792-019-0729-z

**Published:** 2019-11-08

**Authors:** Yuichiro Furutani, Chikashi Hiranuma, Masakazu Hattori, Kenji Doden, Yasuo Hashizume

**Affiliations:** Department of Surgery, Fukui Prefectural Hospital, Chome-8-1 Yotsui, Fukui, Fukui 910-8526 Japan

**Keywords:** Portal venous gas, Conservative therapy, Colon cancer surgery

## Abstract

**Background:**

Portal venous gas has traditionally been considered an inevitable harbinger of death due to its association with bowel necrosis. Recently, an increasing number of cases of portal venous gas have been reported in patients with various clinical conditions and without bowel necrosis. We herein report the case of a patient in whom portal venous gas developed after transverse colon cancer surgery.

**Case presentation:**

A 69-year-old man who had transverse colon cancer underwent insertion of a transanal ileus tube for decompression. Transverse colon resection was performed on the 11th day after the insertion of the transanal ileus tube. The patient had a high fever on the 6th day after the operation. Computed tomography showed portal venous gas over the entire area of the liver and pneumatosis intestinalis in the wall of the ascending colon. There were no signs of anastomotic leakage or bowel necrosis, so we decided to use conservative therapy with fasting and antibiotics. The portal venous gas disappeared on the 19th day after the operation. The patient was discharged in good condition on the 29th day after the operation.

**Conclusions:**

Conservative treatment for portal venous gas is reasonable for patients without signs of anastomotic leakage or bowel necrosis. However, it is important to carefully observe patients with portal venous gas during conservative treatment because portal venous gas may be life-threatening.

## Background

Portal venous gas has traditionally been considered an inevitable harbinger of death due to its association with bowel necrosis [1]. However, the frequent use of computed tomography (CT) has led to an increasing frequency of detection of portal venous gas in patients with various conditions. Recently, an increasing number of cases of portal venous gas have been reported in various patients without bowel necrosis [1]. We herein report the case of a patient with transverse colon cancer in whom portal venous gas developed after transverse colon resection without anastomotic leakage or bowel necrosis.

## Case presentation

**Fig. 1:**
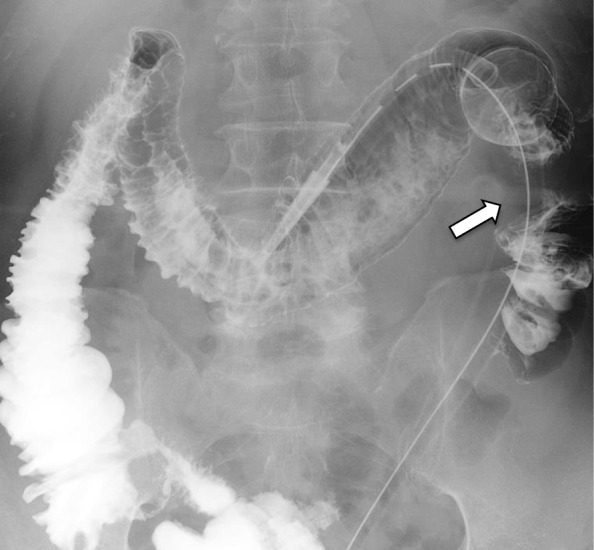
Enema X-ray before operation. Enema X-ray showed light narrowing and a rough mucosal surface in the transverse colon. The arrow shows transverse colon cancer

A 69-year-old man was admitted to our hospital because of ileus. His abdomen was extremely distended. Colonoscopy revealed severe stenosis caused by transverse colon cancer at splenic flexure. We inserted a transanal ileus tube for decompression. Enema X-ray showed transverse colon cancer, a light narrowing and rough mucosal surface from the transverse colon to the ascending colon (Fig. 1). Because of the mild inflammatory response, cefmetazole was administered as an antibiotic for 11 days until surgery. Because the patient’s general findings, abdominal findings, and blood examination findings were improved, transverse colon resection was performed on the 11th day after the insertion of the transanal ileus tube. Pathological findings showed no mucosal necrosis in the transverse colon surrounding the tumor. During the perioperative period, cefmetazole was administered as an antibiotic from the operation day to the first day after operation.

**Fig. 2:**
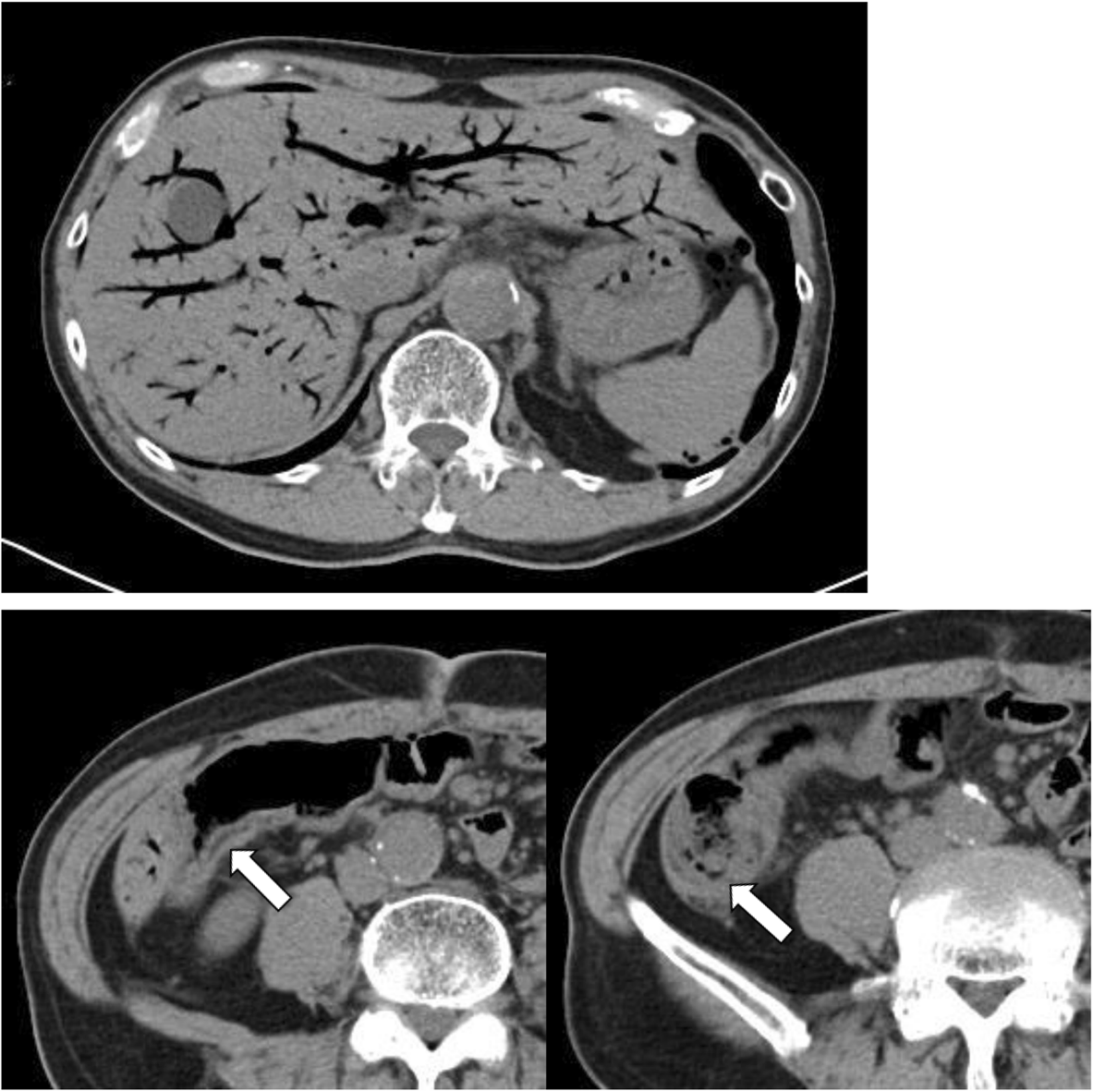
CT 6 days after the operation. CT showed portal venous gas over the entire area of the liver. The arrow shows pneumatosis intestinalis in the thick wall of the ascending colon

**Fig. 3:**
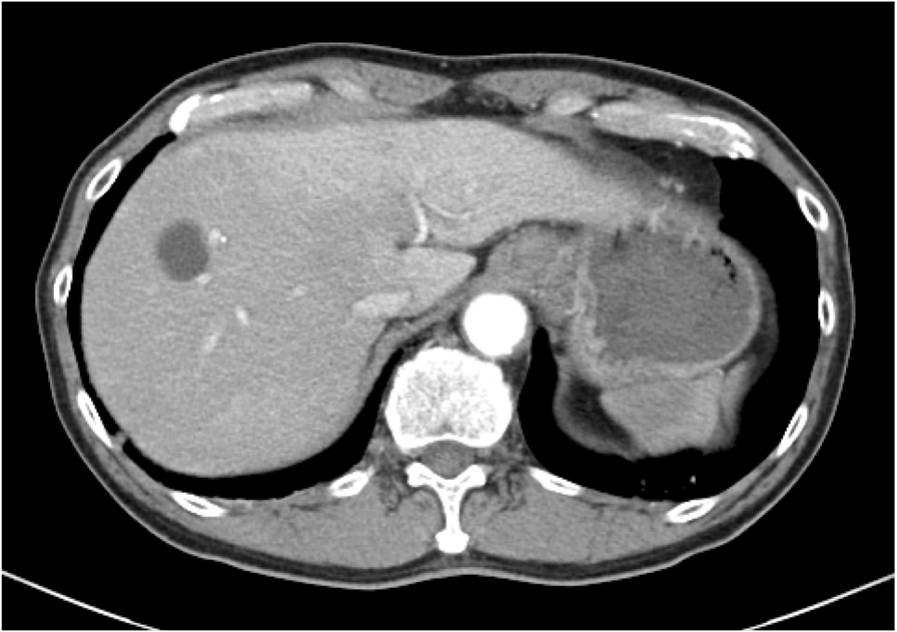
CT 19 days after the operation. CT showed that both hepatic portal gas and pneumatosis intestinalis had disappeared

The patient had a high fever on the 6th day after the operation. CT showed portal venous gas over the entire area in the liver and pneumatosis intestinalis in the thick wall of the ascending colon (Fig. 2). Blood examination showed severe liver damage (Table 1). The patient did not complain of abdominal pain. On examination, the patient’s abdomen was flat and soft. There were no signs of anastomotic leakage or bowel necrosis, so we decided to use conservative therapy with fasting and antibiotics. We selected tazobactam/piperacillin as an antibiotic since the 6th day after the operation, considering gas-forming bacteria. Although the blood was taken for the blood culture on that day, the blood culture was negative on the 14th day after the operation. Gradually, the severe liver damage improved, as assessed through blood examination, since conservative therapy was started. The patient began oral intake on the 10th day after the operation. Finally, CT showed that both the hepatic portal gas and pneumatosis intestinalis had disappeared on the 19th day after the operation (Fig. 3). The patient was discharged from the hospital on the 29th day after the operation.

**Table 1:** Blood examination

WBC	10,700/μL
RBC	350/μL
Hb	10.9 g/dL
Ht	33.2/μL
Plt	25.0 × 10^4^/μL
AST	894 IU/L
ALT	768 IU/L
LDH	1196 IU/L
ALP	1215 IU/L
TB	0.9 mg/dL
BUN	17.5 mg/dL
Cre	0.99 mg/dL
TP	5.9 g/dL
Alb	2.9 g/dL
Na	136 mEq/L
K	4.3 mEq/L
Cl	100 mEq/L
CRP	10.6 mg/dL

*WBC* white blood cell, *RBC* red blood cell, *Hb* hemoglobin, *Ht* hematocrit, *Plt* platelet, *AST* aspartate aminotransferase, *ALT* alanine aminotransferase, *LDH* lactate dehydrogenase, *ALP* alkaline phosphatase, *TB* total bilirubin, *BUN* blood urea nitrogen, *Cre* creatinine, *TP* total protein, *Alb* albumin, *CRP* C-reactive protein

## Discussion

Portal venous gas was first reported in 1955 by Wolfe and Evans [2]; since then, it has traditionally been considered an inevitable harbinger of death. It is known that there are certain causes of portal venous gas [3, 4]. The first cause is increased pressure in the intestinal tract. There are many reasons for increased pressure in the intestinal tract, such as digestive tract dilatation, ulcerative colitis, gastric ulceration, complications of endoscopic procedures, or Crohn’s disease [5]. Thus, gas escapes from the mesenteric circulation into the liver vasculature. The second cause is the abnormal occurrence of gas-forming bacteria such as *Escherichia coli*, *Proteus mirabilis*, *Klebsiella pneumoniae*, and *Clostridium* spp. Mesenteric vascular occlusion, bowel ischaemia, and subsequent bowel necrosis can induce the abnormal occurrence of gas-forming bacteria. The third cause is mucosal damage of the intestinal tract, such as necrosis, inflammation, and ulcers.

In this case, the patient had digestive tract dilatation due to transverse colon cancer before surgery. Therefore, we inserted a transanal ileus tube to reduce digestive tract dilatation and continued inserting a transanal ileus tube for 11 days. We operated on the patient after confirming that his abdomen was sufficiently soft and flat. The patient had portal venous gas on the 6th day after the operation. It is known that obstructive colitis causes mucosal damage in digestive tract dilatation. A preoperative enema X-ray showed light narrowing and a rough mucosal surface from the transverse colon to the ascending colon. Transverse cancer was located at splenic flexure, and the edema of the ascending colon was very little as long as we confirmed by touching during the operation. In addition to it, if the ascending colon was removed until the splenic flexure of the transverse colon, it was judged that the invasion was too much. Overall, we thought that anastomosis was possible, leaving the ascending colon. We selected transverse colon resection instead of right hemicolectomy as the surgical procedure. In fact, there was no evidence of mucosal necrosis in the transverse colon surrounding the tumor, both macroscopically and pathologically. But we believe that the mucosal damage of obstructive colitis could have remained after the operation. Thus, mucosal damage could be the cause of the abnormal occurrence of gas-forming bacteria. In this case, the blood culture was negative. We suspect that the reason is because antibiotics were administered during the perioperative period. Our reflection of this case is that we should have left more time before the operation. It is very difficult to decide when to operate because there are no predictive factors in these patients. It is important not to hurry the operation even if the patient’s general findings, abdominal findings, and blood examination findings are stable.

In our case, the CT scan after the surgery showed portal venous gas in the liver and air in the wall of the remaining ascending colon. Portal venous gas tends to appear more in the anterior segments of the hepatic left lobe and hepatic right lobe than in the posterior segment of the hepatic right lobe [6]. Therefore, in our patient, it was unusual that portal venous gas spread to the entire area of the liver. Although signs of severe liver damage appeared from the blood examination, at the same time, the patient did not complain of abdominal pain. There were no signs of anastomotic leakage or bowel necrosis, so we decided to use conservative therapy with fasting and antibiotics. Griffiths and Gough [7] state that portal venous gas leads to a decrease in blood flow, causing detoxification disorders in Kupffer cells. For that reason, when the number of bacteria increases in the bloodstream, the patient tends to have severe blood poisoning. Although the blood culture was negative on the 14th day after the operation, we selected tazobactam/piperacillin as the antibiotic, considering the previously mentioned gas-forming bacteria.

Since we began the conservative therapy, the severe liver damage improved, and the portal venous gas disappeared gradually. We were able to avoid surgery for the portal venous gas and pneumatosis intestinalis in the wall of the ascending colon. Although portal venous gas can be managed with conservative therapy in some cases [8], considering the differential diagnosis is important. This is because similar CT findings are reported in patients with anastomotic leakage or bowel necrosis. When portal venous gas appears in any situation, we should consider not only the CT findings but also the patient’s total condition, including vital signs, abdominal findings, and blood examination findings. It is important to carefully observe patients with portal venous gas in conservative treatment because portal venous gas may be life-threatening.

## Conclusion

Portal venous gas can appear after obstructive colitis without anastomotic leakage or bowel necrosis. Portal venous gas alone is not a surgical indication, and we should consider the patient’s total condition, including vital signs, abdominal findings, and blood examination findings. Conservative treatment against portal venous gas can be reasonable for patients without signs of anastomotic leakage or bowel necrosis.

## Abbreviation

CT: Computed tomography
